# Prolyl Hydroxylase Domain-2 Inhibition Improves Skeletal Muscle Regeneration in a Male Murine Model of Obesity

**DOI:** 10.3389/fendo.2017.00153

**Published:** 2017-07-05

**Authors:** Indranil Sinha, Dharaniya Sakthivel, Benjamin A. Olenchock, Carla R. Kruse, Jeremy Williams, David E. Varon, Jessica D. Smith, Arin L. Madenci, Kristo Nuutila, Amy J. Wagers

**Affiliations:** ^1^Division of Plastic Surgery, Brigham and Women’s Hospital, Boston, MA, United States; ^2^Harvard Medical School, Boston, MA, United States; ^3^Harvard Stem Cell Institute, Cambridge, MA, United States; ^4^Division of Cardiovascular Medicine, Brigham and Women’s Hospital, Boston, MA, United States; ^5^University of California San Francisco, San Francisco, CA, United States; ^6^Joslin Diabetes Center, Boston, MA, United States

**Keywords:** obesity, hypoxia, prolyl hydroxylase domain enzymes, skeletal muscle regeneration, vascular endothelial growth factor

## Abstract

Obesity leads to a loss of muscle mass and impaired muscle regeneration. In obese individuals, pathologically elevated levels of prolyl hydroxylase domain enzyme 2 (PHD2) limit skeletal muscle hypoxia-inducible factor-1 alpha and vascular endothelial growth factor (VEGF) expression. Loss of local VEGF may further impair skeletal muscle regeneration. We hypothesized that PHD2 inhibition would restore vigorous muscle regeneration in a murine model of obesity. Adult (22-week-old) male mice were fed either a high-fat diet (HFD), with 60% of calories derived from fat, or a regular diet (RD), with 10% of calories derived from fat, for 16 weeks. On day 5 following cryoinjury to the tibialis anterior muscle, newly regenerated muscle fiber cross-sectional areas were significantly smaller in mice fed an HFD as compared to RD, indicating an impaired regenerative response. Cryoinjured gastrocnemius muscles of HFD mice also showed elevated PHD2 levels (twofold higher) and reduced VEGF levels (twofold lower) as compared to RD. Dimethyloxalylglycine, a cell permeable competitive inhibitor of PHD2, restored VEGF levels and significantly improved regenerating myofiber size in cryoinjured mice fed an HFD. We conclude that pathologically increased PHD2 in the obese state drives impairments in muscle regeneration, in part by blunting VEGF production. Inhibition of PHD2 over activity in the obese state normalizes VEGF levels and restores muscle regenerative potential.

## Introduction

Skeletal muscle maintenance and recovery from injury rely on robust endogenous repair mechanisms, which become impaired with obesity. Diminished capacity of muscle regeneration may contribute to the loss of lean muscle mass often seen in obese diabetic patients (1) and can result from a myriad of factors, including insulin resistance, impaired metabolism, and chronic inflammation (2). Multiple studies suggest that muscle wasting in these patients can be severe, especially for those above the age of 60 (3–5). Mice fed a high-fat diet (HFD) similarly exhibit impaired muscle regeneration following injury, as well as a reduced number of muscle satellite cells (6, 7). The mechanisms underlying obesity-associated loss of skeletal muscle mass and regenerative potential are not entirely known.

Upregulation of the hypoxia-inducible factor-1 alpha (HIF-1α) and induction of vascular endothelial growth factor (VEGF) have been suggested as critical components for promoting proper muscle regeneration after injury. Loss of HIF-1α decreases skeletal muscle endurance (8), and the loss of VEGF in transplanted muscle stem cells impairs their engraftment and myofiber regeneration following transplantation (9). HIF-1α is dysregulated in diabetic patients, resulting in a reduction of local HIF-1α bioavailability, low VEGF activity, poor angiogenesis following injury, and impaired wound healing (10, 11). In addition, relative tissue hypoxia in obesity can itself lead to increased systemic inflammation and loss of insulin sensitivity, both of which exert deleterious effects on muscle regeneration (12, 13).

Interestingly, deletion of the hypoxia-response element in the VEGF promoter results in neural degeneration and loss of lean muscle mass (14). In patients with obesity, limited VEGF expression is implicated in impaired skeletal muscle regeneration, although the mechanism(s) underlying this effect is unclear (15). The etiology that results in coincidence of lower HIF-1α and VEGF levels occurring with obesity, especially in tissue injury responses, also remains unknown.

Previous studies suggest that the prolyl hydroxylase domain (PHD) family of enzymes, which regulate HIF-1α and VEGF activity, are pathologically increased in models of obesity and type 2 diabetes mellitus (T2DM) (16, 17). PHDs are active in normoxic conditions and function to degrade HIF-1α and inhibit the hypoxia-response pathway (18). Multiple PHD isoforms exist, with prolyl hydroxylase domain enzyme 2 (PHD2) being the predominant form in skeletal muscle (19, 20). In normoxic conditions, PHD-mediated hydroxylation of proline residues within the HIF-1α protein results in its binding to the Von Hippel–Lindau tumor-suppressor protein, which interacts with the protein elongin C and thereby recruits an E3 ubiquitin-protein ligase that targets HIF-1α for ubiquitination and degradation by the proteasome. In hypoxic conditions, PHD2 is normally inactivated, and HIF-1α and HIF-2α activities increase (21).

Silencing of EGLN1 (the gene encoding PHD2) locally in skeletal muscle is protective from ischemia–reperfusion injury and promotes neovascularization, mediated by VEGF activity (22, 23). Likewise, pharmacologic inhibition of PHD2 increases VEGF transcription, with corollary enhancement of angiogenesis shown in a model of T2DM wound healing (24). In addition, mice with decreased PHD1 or PHD3 activity similarly exhibit protection against hind limb ischemia and preservation of muscle mass following ischemic insult (21, 25–27). However, this PHD2/HIF-1α/VEGF pathway has yet to be assessed in the context of induced skeletal muscle injury and repair. The present study was therefore designed to evaluate the involvement of PHDs in muscle regeneration in a murine obesity model and to determine whether pharmacologic inhibition of PHD2 can improve muscle regenerative responses in this context.

## Animals and Methods

### Animals

The Institutional Animal Care and Use Committee of Brigham and Women’s Hospital and Joslin Diabetes Center approved all animal procedures described (2016N000375). Twenty-two-week-old male C57Bl/6J mice fed a control diet [regular diet (RD)], *ad libitum*, for 16 weeks and diet-induced obesity male C57BL/6J mice fed an HFD, *ad libitum* (60% calories from fat), were obtained from pathogen-free breeding colonies (The Jackson Laboratory, Bar Harbor, ME, USA). These mice exhibit obesity and are prediabetic, with impaired glucose tolerance tests and decreased insulin sensitivity.^1^ For maintenance of diet, RD (D12450Bi) and HFD (D12492) were obtained from Research Diets (New Brunswick, NJ, USA), which contained 10 and 60% calories from fat, respectively. To determine the *in vivo* therapeutic efficacy of PHD2 inhibition in promoting skeletal muscle regeneration, mice continuously fed either an RD or an HFD were injected intraperitoneal (IP) with a 160 mg/kg dose of dimethyloxalylglycine (DMOG) (Sigma-Aldrich, St. Louis, MO, USA) prepared in saline vehicle (0.9% sodium chloride) for 5 days, starting 1 day prior to cryoinjury. As a control, another group of mice received IP saline vehicle injections only.

### Cryoinjury of Muscle and Quantification of Cross-sectional Area of Regenerating Myofibers

For cryoinjury, mice were anesthetized, and dry ice was applied directly to the exposed tibialis anterior (TA) and gastrocnemius muscles for 5 s. The skin incision was closed with 4–0 Prolene suture (Ethicon Inc., Somerville, NJ, USA) immediately after injury. This procedure generates a reproducible injury in the muscle with a discrete border between uninjured and injured muscle (28, 29). Injured muscles were allowed to recover for 5 days prior to mouse euthanasia and muscle harvest. For quantification of regenerating myofiber size after cryoinjury, a series of images were taken spanning the entire regenerating area in cross section (CSA); the sizes of 10 regenerating myofibers (identified by their centrally located nuclei) were measured in each image using ImageJ software (RRID: SCR_003070), which collectively resulted in a total of approximately 100 myofiber sizes measured for each animal.

### Histology and Immunohistochemisty

Harvested TA muscles were fixed in 10% neutral buffered formalin solution (Sigma-Aldrich, St. Louis, MO, USA) for 48 h and transferred to 70% ethanol thereafter. Fixed tissues were embedded in paraffin blocks, and 8 µm sections were cut with a microtome (Microm HM 550, Thermo Fisher Scientific, Waltham, MA, USA) and mounted. Deparaffinization and rehydration were performed using xylene (Fisher Scientific, First Lawn, NJ, USA) and a series of graded ethanol solutions (100, 95, 75, and 50%). Sections were then stained with Gill’s 3 hematoxylin (Thermo Fisher Scientific, Cheshire, UK) and eosin (Sigma-Aldrich, St. Louis, MO, USA). Images were acquired using a DS-Fi1 camera and Nikon Eclipse E400 microscope (Nikon Corporation, Tokyo, Japan).

For immunohistochemical analysis, sections were first deparaffinized. Epitope retrieval was performed using Leica enzyme retrieval agents for 10 min. Sections were then incubated with primary anti-CD31 (Biocare Medical, Concord, CA, USA) at 1:50 overnight followed by incubation with secondary goat anti-rat IgG HRP antibody at 1:50 (Millipore, Darmstadt, GER). Slides were developed using 3-diaminobenzidine chromogen and counterstained with hematoxylin. For quantification of CD31 positive vessels, serial sections were taken spanning the CSA, and the number of CD31 positive-stained capillaries was counted per high power field (200× magnification). A minimum of 10 distinct sections were analyzed per sample.

### Real-time PCR

The gastrocnemius muscle was harvested and homogenized using a gentleMACS dissociator (Miltenyi Biotec, Cambridge, MA, USA) for RNA extraction using Trizol/chloroform. One microgram of total RNA was reverse transcribed in a 20 µL reaction using the Super Script III First-Strand System (Invitrogen, Carlsbad, CA, USA). Hypoxia arrays were performed utilizing the RT^2^ qPCR Hypoxia Array (SABiosciences, Frederick, MD, USA). Arrays were performed using a StepOnePlus Real-time PCR (Applied Biosystems, Foster City, CA, USA) with denaturation at 95°C (10 min) followed by 40 cycles of 95°C (15 s) and 60°C (1 min). The *C*_t_ values were then analyzed using SABioscience’s Data Analysis Web Portal, and the resulting fold changes were normalized to housekeeping genes (Actb, B2m, Gapdh, Gusb, and Hsp90ab1). Fold difference values against control group >2 were considered as upregulated genes and <0.5 were considered as downregulated genes. All array samples were confirmed to have genomic DNA amplification *C*_t_ levels greater than 35, suggesting minimal or no contamination.

### Western Blotting and ELISAs

Whole muscle lysates were isolated from the gastrocnemius muscle as previously described (NE-PER Nuclear and Cytoplasmic Extraction Reagent Kit, Pierce, Rockford, IL, USA) (30). Following subcellular fractionation, PHD2 and VEGF were analyzed in the cytoplasmic fraction, and both HIF-1α and HIF-2α were assessed in cytoplasmic and nuclear fractions through the use of ELISA and immunoblotting. In brief, proteins were separated on a 4–12% SDS-polyacrylamide gel with MOPS SDS Running Buffer (Novex-Life Technologies, Carlsbad, CA, USA) at 150 V. The gel was transferred onto an Immuno-Blot PVDF Membrane using the iBlot2 Dry Blotting System (Thermo Fisher Scientific, Cheshire, UK), for 7 min. Membranes were then blocked in blocking solution (Life Technologies, Frederick, MD, USA) for 1 h at room temperature then probed overnight at 4°C with constant shaking. Antibodies utilized are listed in Table 1. Following three 5 min washes in TBS-T buffer, membranes were incubated in anti-rabbit IgG HRP-linked (Cell Signaling Technology, Danvers, MA, USA, Cat# 7074 RRID: AB_2099233) secondary antibodies. All antibodies were diluted in blocking buffer. For immunodetection, membranes were washed three times with TBS-T buffer, incubated with ECL solutions per manufacturer’s specifications (Amersham Biosciences, Pittsburgh, PA, USA), and exposed to Hyperfilm ECL. The membranes were stripped and reprobed with an antibody recognizing α-tubulin for normalization as a control. Band intensities were determined using ImageJ software.

**Table 1:** List of antibodies.

Protein	Species	Dilution	Company
PHD2	Rabbit	1:1,000	Cell Signaling Technologies RRID: AB_10561316
HIF-2α	Rabbit	1:1,000	Novus RRIS: AB_10002593
VEGFa	Rabbit	1:1,000	Abcam RRID: AB_2212642
α-Tubulin	Rabbit	1:1,000	Cell Signaling Technologies RRID: AB_2210548
CD31 (PECAM-1)	Rat	1:50	Biocare Medical RRID: –

### Statistical Analysis

Data are presented as the mean and SD. Statistical comparisons for normally distributed data were performed using appropriate tests. Results comparing two different groups were assessed for statistical significance using Student’s *t*-test. Results comparing more than two groups were assessed by one-way ANOVA with Tukey’s multiple comparison test (GraphPad Prism, GraphPad Software Inc., San Diego, CA, USA, RRID: SCR_002798). For statistical analyses of distribution and average regenerating myofiber sizes in injured muscles, *p*-values were calculated by Kruskal–Wallis test and adjusted, if necessary, by the step-down-Bonferroni method as previously described (29). Investigators were blinded to experimental group assignment for outcome assessment. Statistical significance was accepted at *p* < 0.05.

## Results

### Obesity Is Associated with Decreased Skeletal Muscle Regeneration *In Vivo*

**Figure 1:**
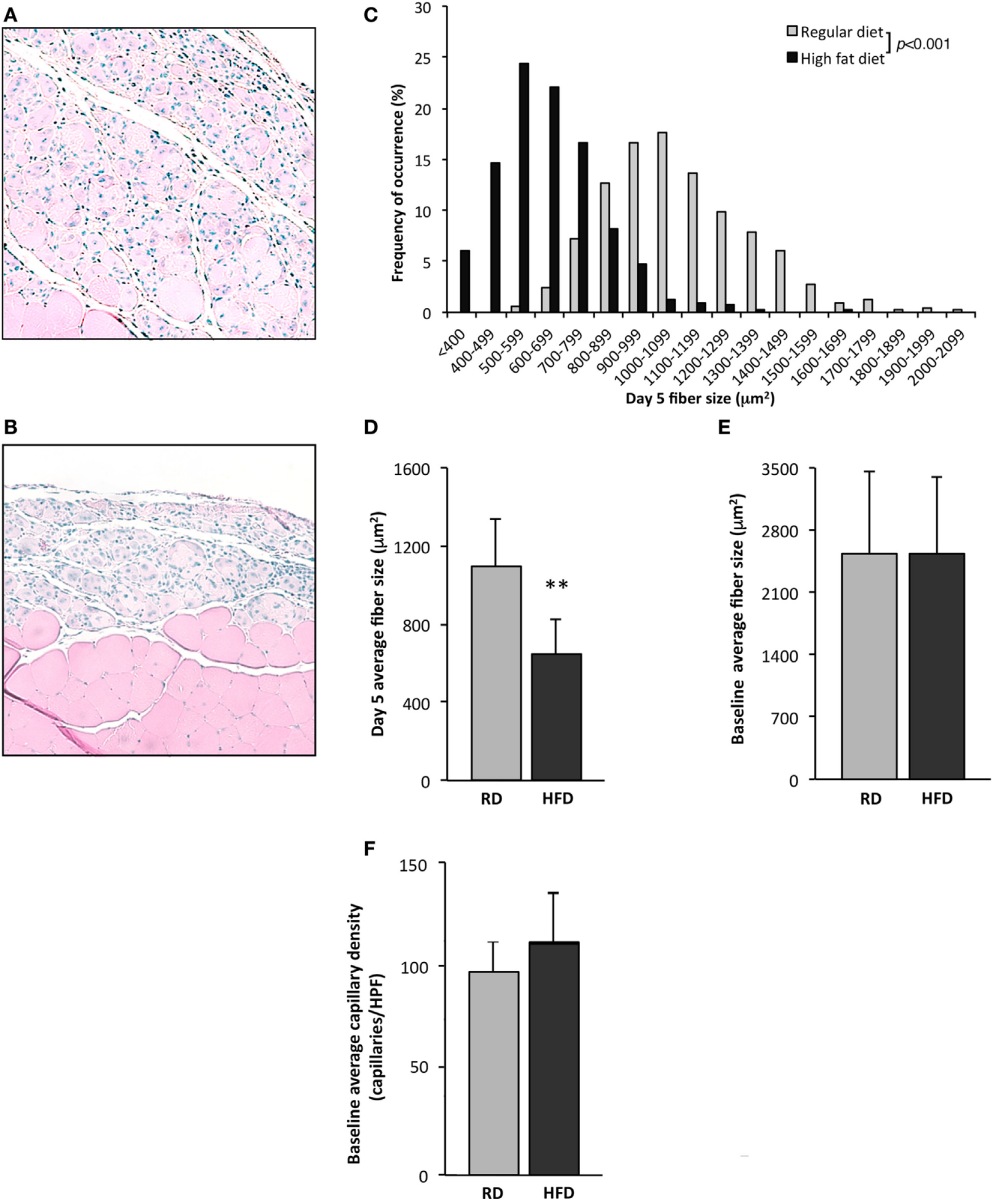
Exposure to a high-fat diet limits skeletal muscle regeneration. Twenty-two-week old mice fed a high-fat diet (HFD) for 16 weeks were compared to similarly aged mice on a regular diet (RD). Quantification of uninjured fiber size and regenerating (centrally nucleated) myofiber size in tibialis anterior muscles 5 days after cryoinjury for control mice receiving an RD (*n* = 5 mice) **(A)** was compared with mice fed an HFD (*n* = 5) **(B)**. Representative figures for regenerating fibers on day 5 following injury as seen on H&E stained sections are presented in this figure and demonstrate significant differences in regeneration. Regenerating fiber size at day 5 following injury, fiber size (binned by 100 µm^2^ increments) **(C)**, or as average fiber cross-sectional area **(D)**, was significantly larger in the group fed RD as compared to HFD. There is no difference in cross-sectional fiber area of uninjured muscle (*n* = 5) **(E)**, or capillary density (*n* = 4) **(F)**. *p*-Values determined by Kruskal–Wallis test with step-down Bonferroni method for panel **(C)**. *p*-Values otherwise calculated by Student’s *t*-test (**p < 0.01).

To evaluate deficits in skeletal muscle regeneration that accompany obesity, mice fed either an RD or HFD were subjected to hindlimb muscle cryoinjury. Mice on an HFD exhibited increased weight (48 ± 3 vs. 31 ± 4 g, *p* < 0.001, *n* = 10 per group) and elevated fasting serum glucose levels (179 ± 75 vs. 111 ± 27 mg/dL, *p* < 0.001, *n* = 10 per group), as compared to RD controls. On day 1 following cryoinjury, performed to induce skeletal muscle regeneration, muscles showed scant regenerating fibers in either group. Regenerating fibers were detected in both groups at day 5 after injury, however, fiber CSA was significantly smaller in the HFD group as compared to RD group (643 ± 183 vs. 1,092 ± 246 µm^2^, *p* < 0.01, *n* = 5 per group, Figures 1A–D). More importantly, these differences showed divergent regenerative responses to muscle injury, as no differences were noted in myofiber CSA in age-matched uninjured mice following 16 weeks of HFD or RD (2,531 ± 860 vs. 2,542 ± 912 µm^2^, *n* = 5 per group, respectively, Figure 1E). In addition, there were no significant baseline differences noted in the capillary density of mice fed HFD (114 ± 24 capillaries/HPF) as compared to RD (99 ± 15 capillaries/HPF, *n* = 4 per group, Figure 1F).

### Hypoxia Signaling Pathway Is Dysregulated in Obese Mice following Cryoinjury to Skeletal Muscle

Following cryoinjury, hindlimb muscles were harvested on post-procedure day 5 and subjected to Hypoxia PCR Array profiling. The expression of 84 genes relevant to hypoxia signaling was assessed. Differentially regulated genes are listed in Table 2 (increased expression in HFD mice) and Table 3 (decreased expression in HFD mice). All genes with fold change >2 (absolute value) are listed. EGLN 1 (PHD2) and EGLN 2 (PHD1) transcription levels were 2- and 2.7-fold lower, respectively, in mice fed an HFD following injury. HIF-1α and HIF-2α were not differentially regulated between the two groups. VEGF(a) levels were 2.8-fold lower by array profiling in the regenerating gastrocnemius of mice fed an HFD following cryoinjury.

**Table 2:** Genes upregulated in skeletal muscle of mice fed high-fat diet vs. regular diet on day 5 following cryoinjury.

Gene (symbol)	Fold change
Lysyl oxidase (Lox)	2.4
Serine/threonine-protein kinase (Atr)	2.4
Carbonic anhydrase 9 (Car9)	2.3
Pyruvate dehydrogenase kinase (Pdk1)	2.3
β-Glucuronidase (Gusb)	2.1

**Table 3:** Genes downregulated in skeletal muscle of mice fed high-fat diet vs. regular diet on day 5 following cryoinjury.

Gene (symbol)	Fold change
Vascular endothelial growth factor a (VEGFa)	2.8
EGL nine 2 (EGLN2)	2.7
Triosephosphate isomerase 1 (Tpi 1)	2.2
Glucose phosphate isomerase 1 (Gpi1)	2.1
EGL nine 1 (EGLN1)	2.0

**Figure 2:**
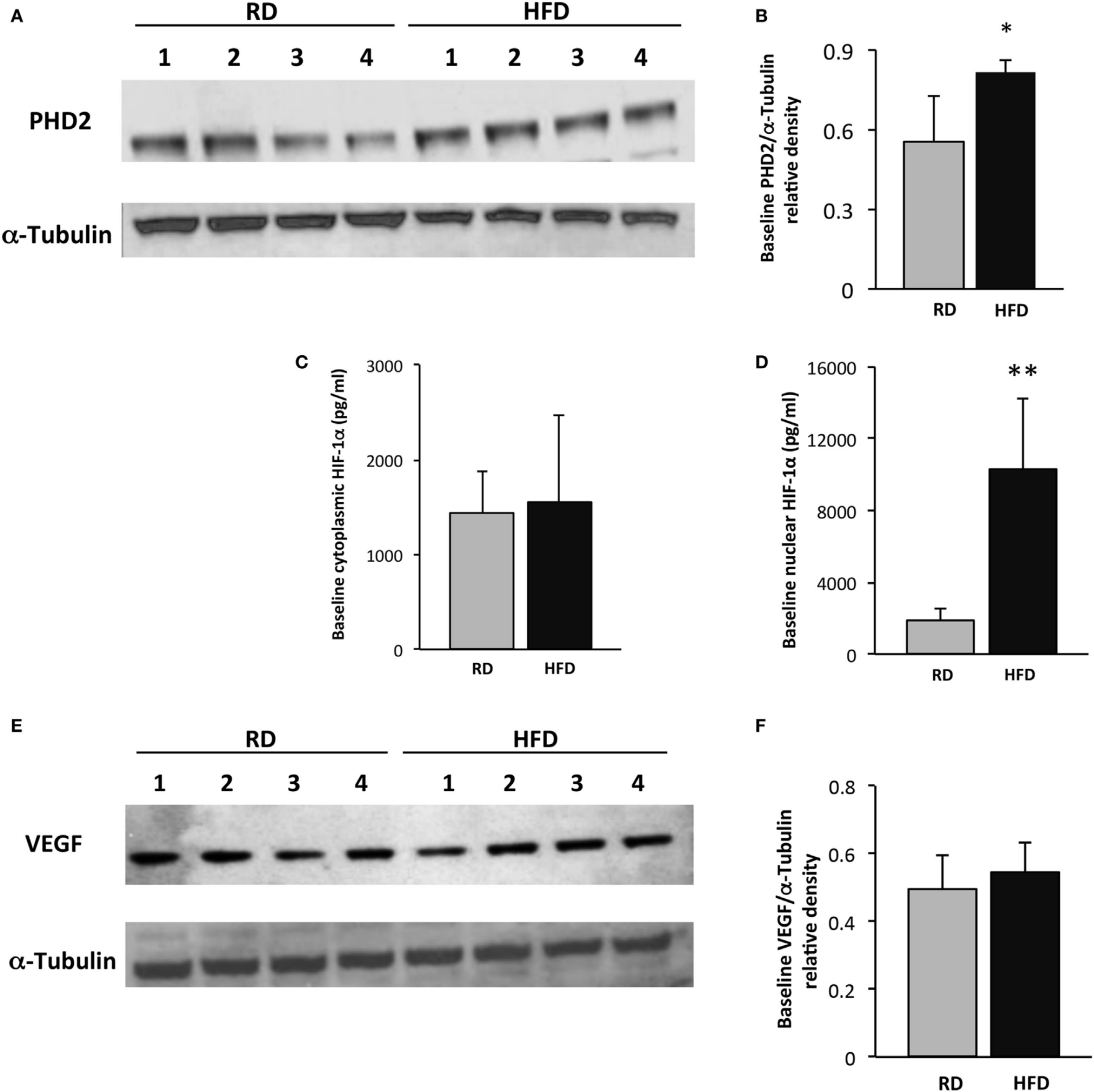

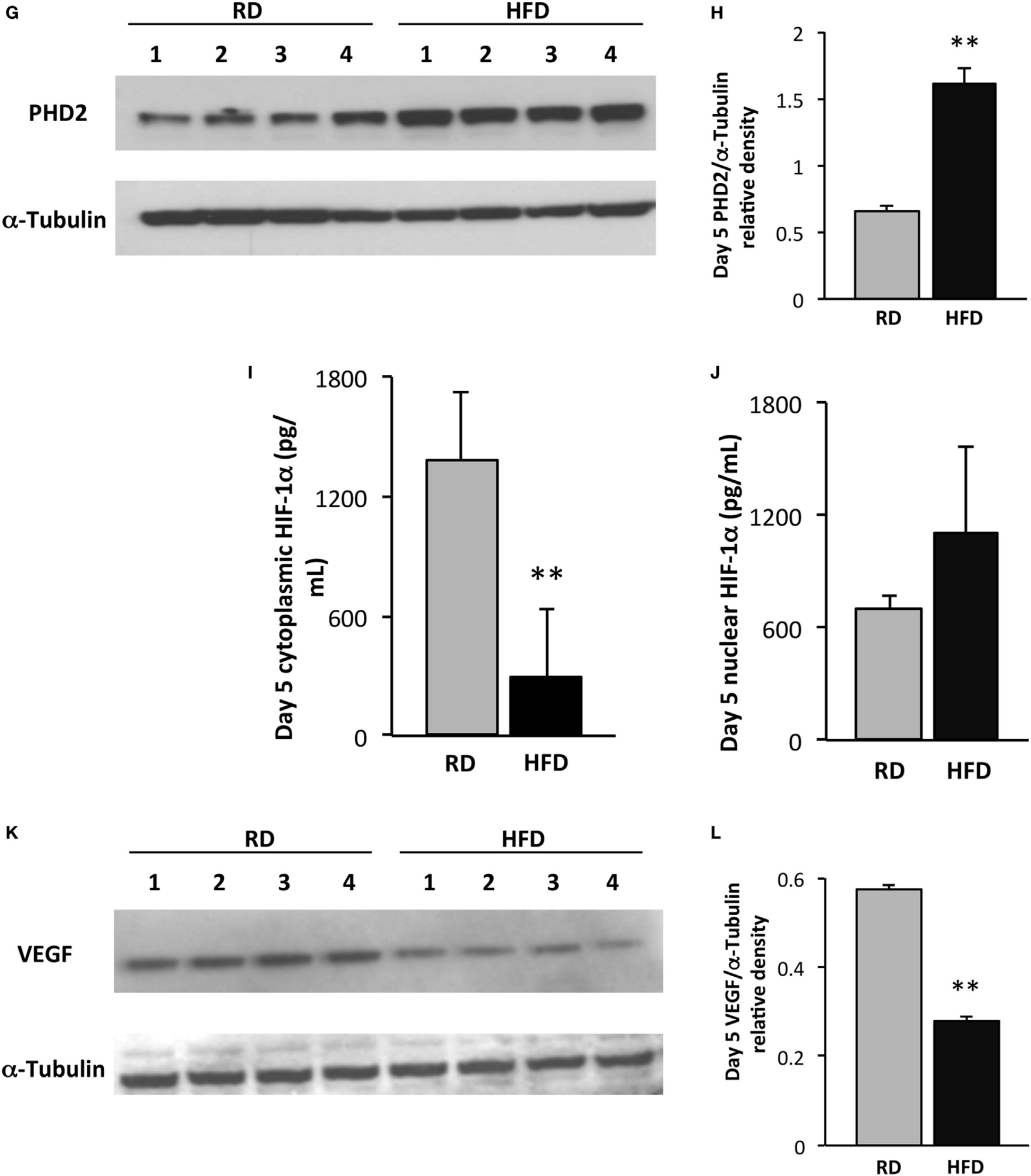
Skeletal muscle vascular endothelial growth factor (VEGF) expression is restrained following injury in an obesity model. Western blot of lysates from gastrocnemius muscle from control (*n* = 4) and high-fat diet (HFD) (*n* = 4) mice shows elevated prolyl hydroxylase domain enzyme 2 (PHD2) levels in HFD mice at baseline **(A)**. Quantification of PHD2 band intensities is depicted in panel **(B)**. ELISA demonstrates similar levels of cytoplasmic hypoxia-inducible factor-1 alpha (HIF-1α) at baseline **(C)**, but a marked increase in nuclear levels of HIF-1α **(D)**. Western blot of lysates from gastrocnemius muscle from mice fed either HFD or regular diet (RD) exhibits similar levels of VEGF at baseline **(E)**. Quantification of VEGF band intensities is depicted in panel **(F)**. On day 5 following injury, immunoblotting using lysates from gastrocnemius muscle demonstrates significantly elevated PHD2 levels in mice fed HFD vs. RD **(G)**. Quantification of PHD2 band intensities is depicted in panel **(H)**. ELISA demonstrates a decrease in skeletal muscle cytoplasmic HIF-1α in mice fed HFD as compared to RD **(I)**, but not in the nuclear subfraction **(J)**. Western blots of lysates from gastrocnemius muscle from control (*n* = 4) and DIO (*n* = 4) mice demonstrate a corollary decrease in VEGF levels in HFD vs. RD on day 5 following cryoinjury **(K)**. Quantification of VEGF band intensities is depicted in panel **(L)**. α-Tubulin is shown as a loading control for all immunoblots (*p < 0.05, **p < 0.01).

Prolyl hydroxylase domain enzyme 2 protein levels, detected by immunoblotting, were slightly higher at baseline in the hindlimb muscles of mice fed an HFD as compared to RD [0.81 ± 0.05 vs. 0.55 ± 0.17 arbitrary units (au), *p* = 0.02, *n* = 4 per group]. Nuclear HIF-1α levels were significantly higher in mice fed an HFD as compared to RD by ELISA (10,313 ± 3,956 vs. 1,829 ± 657 pg/mL, respectively, *p* < 0.01, *n* = 5 per group), but there were no differences detected in VEGF levels by immunoblotting (Figures 2A–F). By day 5 following injury, PHD2 protein levels were more than twofold higher in mice fed an HFD as compared to RD mice (1.6 ± 0.12 vs. 0.66 ± 0.04 au, respectively, *p* < 0.001, *n* = 4, Figures 2G,H). HIF-1α levels were concomitantly lower in the cytoplasmic fraction of hindlimb muscle from mice fed HFD as compared to RD (297 ± 344 vs. 1,387 ± 342 au, *p* < 0.01, *n* = 5 per group) by ELISA (Figure 2I), but absolute value levels were unchanged in the nuclear fraction (Figure 2J). In correlation with these changes in PHD2 and HIF-1α, VEGF levels were twofold lower in HFD mice vs. RD mice (0.28 ± 0.01 vs. 0.58 ± 0.01 au, *p* < 0.01, *n* = 4 per group, Figures 2K,L).

#### Pharmacologic Inhibition of PHD2 Improves Skeletal Muscle Regeneration *In Vivo*

**Figure 3:**
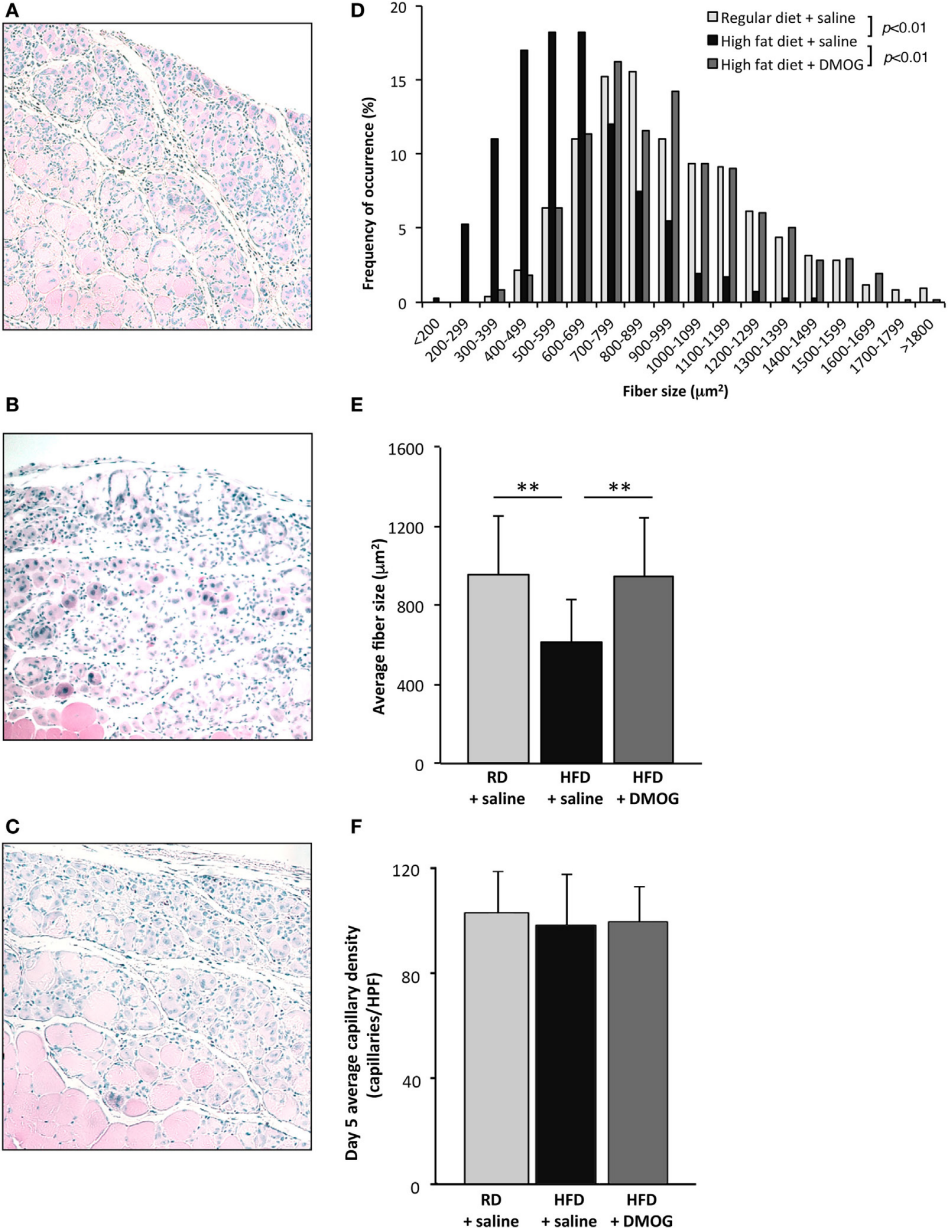
Dimethyloxalylglycine (DMOG) partially reverses obesity-associated loss of skeletal muscle regeneration. Representative H&E staining of muscle sections taken 5 days after cryoinjury from control diet mice (*n* = 5), high-fat diet (HFD) mice (*n* = 5), or HFD mice receiving DMOG (*n* = 5) for 1 day prior to and 4 days following injury **(A,B,C)**. HFD mice remained on HFD following injury. Quantification of regenerating (centrally nucleated) myofiber size at day 5 after cryoinjury suggests that HFD mice receiving DMOG treatment (*n* = 5) exhibit greater skeletal muscle regeneration than HFD mice treated with saline (*n* = 5) and similar to control mice treated with saline (*n* = 5). Data presented as a histogram of fiber size **(D)** or average fiber cross-sectional area **(E)**. There were no differences detected in capillary density **(F)**. *p*-Values were calculated by Kruskal–Wallis test and step-down Bonferroni method for panels **(D,E)** (*p < 0.05, **p < 0.01). Data represented as mean ± SD. **(E)** Scale bars = 100 µm.

To evaluate the effects of pharmacologic PHD2 inhibition, starting 1 day prior to cryoinjury, RD and HFD mice were subjected to either IP injections of saline (vehicle control) or DMOG (PHD2 inhibitor). Consistent with our earlier results, newly regenerated muscle fiber CSA, measured at day 5 after injury, was significantly smaller in saline control mice fed an HFD as compared to RD diet (613 ± 219 vs. 952 ± 299 µm^2^, respectively, *p* < 0.01, *n* = 5 per group, Figures 3A,B,D,E). DMOG treatment of mice fed an HFD significantly increased regenerating fiber size as compared to saline treatment (949 ± 292 vs. 613 ± 219 µm^2^, respectively, *p* < 0.01, *n* = 5 per group, Figures 3B–E). Following injury, there were no differences noted in capillary density between mice fed RD and treated with vehicle (103 ± 16 capillaries/HPF), mice fed HFD and treated with vehicle (98 ± 19 capillaries/HPF), or mice fed HFD and treated with DMOG (99 ± 14 capillaries/HPF) (Figure 3F). DMOG treatment did not increase regenerating fiber size (Figures S1A–D in Supplementary Material) or capillary vessel density (Figure S1E in Supplementary Material) in RD mice as compared to saline control.

### PHD2 Pharmacologic Inhibition Restores Skeletal Muscle VEGF Signaling following Injury

**Figure 4:**
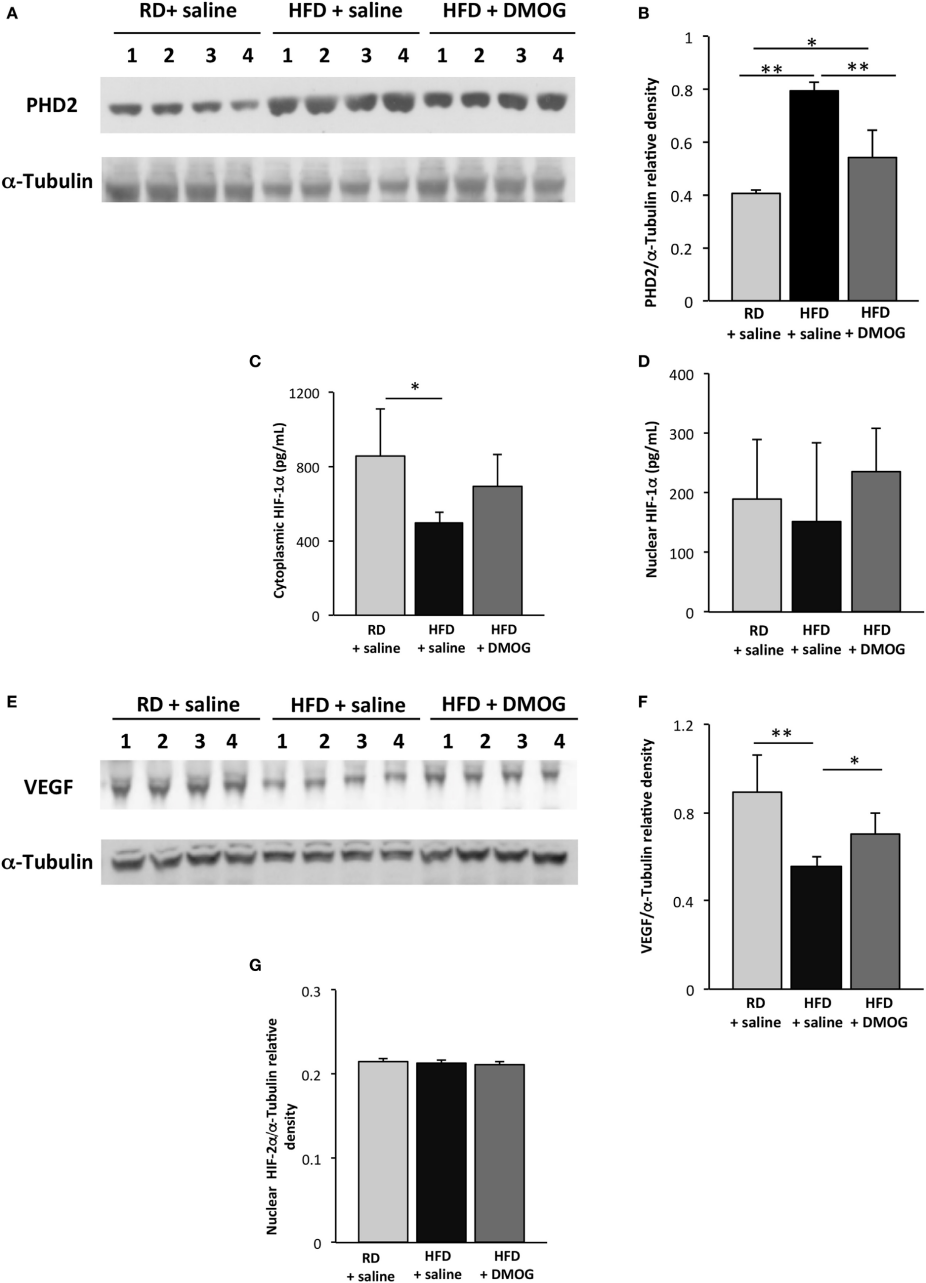
Dimethyloxalylglycine (DMOG) treatment improves hypoxia signaling in skeletal muscle following injury. Immunoblotting lysates from gastrocnemius muscle from mice fed regular diet (RD) with either intraperitoneal injection of DMOG or saline vehicle (*n* = 4 per group) were compared to mice fed high-fat diet (HFD) and receiving DMOG or saline vehicle (*n* = 4 per group). Injections were performed daily and started 1 day prior to and 4 days following cryoinjury. In mice fed HFD, prolyl hydroxylase domain enzyme 2 (PHD2) levels were lessened with DMOG administration **(A)**. Quantification of PHD2 band intensities is depicted in panel **(B)**. ELISA demonstrates cytosolic HIF-1α levels are lower in mice fed HFD on day 5 following injury as compared to RD-fed mice, and there is a trend toward increased cytoplasmic HIF-1α with DMOG treatment **(C)**. There are no significant differences in any group in levels of nuclear HIF-1α **(D)**. Similarly, Western blot of lysates from gastrocnemius muscle demonstrates decreased skeletal muscle vascular endothelial growth factor (VEGF) levels following injury, which is significantly increased with DMOG administration **(E)**. Quantification of VEGF band intensities is depicted in panel **(F)**. Nuclear HIF-2α does not vary between the groups, with or without DMOG supplementation **(G)**. α-Tubulin is shown as a loading control for all immunoblots (*p < 0.05, **p < 0.01). *p*-Values calculated by Student’s *t*-test.

On day 5 following cryoinjury, PHD2 levels were significantly higher in HFD mice treated with saline as compared to RD mice, determined by Western blot analysis (0.79 ± 0.03 vs. 0.41 ± 0.01 au, respectively, *p* < 0.001, *n* = 4 per group, Figures 4A,B). Treatment of HFD mice with DMOG resulted in lower PHD2 levels as compared to HFD mice treated with saline (0.54 ± 0.10 vs. 0.79 ± 0.03 au, *p* < 0.01), whereas no changes were seen in PHD2 levels in hindlimb skeletal muscle in RD mice treated with either saline or DMOG (Figures S2A,B in Supplementary Material).

Cytoplasmic HIF-1α protein levels were significantly reduced in HFD mice treated with saline as compared to RD-fed mice (495 ± 60 vs. 853 ± 257 pg/mL, respectively, *p* < 0.05, *n* = 5 per group, Figure 4C), but as before, levels did not differ in the nuclear fraction as seen in Figure 4D. Treatment of HFD mice with DMOG resulted in a trend toward increased skeletal muscle cytoplasmic HIF-1α following injury as compared to those treated with a saline vehicle (694 ± 167 vs. 495 ± 60 pg/mL, *p* = 0.06, *n* = 5 per group) at day 5 following cryoinjury. DMOG treatment in RD-fed mice did not increase cytoplasmic or nuclear HIF-1α expression in hindlimb muscles as compared to saline control (Figures S2C,D in Supplementary Material).

On day 5 following injury, VEGF protein levels were significantly reduced in HFD mice treated with saline as compared to RD-fed mice (0.56 ± 0.05 vs. 0.89 ± 0.17 au, respectively, *p* < 0.01, *n* = 4 per group, Figures 4E,F). Treatment of HFD mice with DMOG resulted in increased skeletal muscle VEGF levels at day 5 following cryoinjury as compared to mice treated with saline (0.70 ± 0.09 vs. 0.56 ± 0.05 au, *p* = 0.03). DMOG treatment did not increase hindlimb skeletal muscle VEGF expression as compared to saline control (Figures S2E,F in Supplementary Material). HIF-2α levels in the nuclear fraction did not vary between the HFD- and RD-fed groups, with or without DMOG treatment (Figure 4G). HIF-2α was undetectable in the cytoplasmic fraction.

## Discussion

Obesity is associated with impaired skeletal muscle regeneration. Our study demonstrates that PHD2 is increased in skeletal muscle following injury in obese mice and is correlated with decreased HIF-1α and VEGF expression. Fascinatingly, PHD2 inhibition with DMOG ameliorates the skeletal muscle regenerative defects that occur with obesity with a corollary increase in HIF-1α and VEGF levels. These data demonstrate a potential role of PHD2 in skeletal muscle regeneration and suggest that the hypoxia pathway may be a potential therapeutic target to limit obesity-associated myopathy and skeletal muscle loss. Despite pathologic PHD2 upregulation following injury, there were no differences noted in skeletal muscle capillary density, suggesting that PHD2 may be working directly on skeletal muscle to exert its effects.

Oxygen sensor PHDs play important roles in the regulation of HIF-1α and VEGF but can become pathologically elevated in a model of obesity (31). In general, PHD inhibition can lead to HIF-1α or HIF-2α stabilization and nuclear translocation, where HIF functions as a transcription factor and can promote VEGF expression and preserve muscle function (32, 33). The present study demonstrates that PHD2 is increased in hindlimb skeletal muscle following injury, and is correlated with lower levels of HIF-1α and VEGF expression and impaired skeletal muscle regeneration, without any changes observed in HIF-2α expression. However, the exact mechanisms regarding hypoxia pathway dysregulation in obesity are poorly understood (34–36). Interestingly, EGLN 1, the gene encoding PHD2, was transcriptionally down-regulated in mice fed HFD following injury. The regulation of PHDs is complex and may depend on α-ketoglutarate concentrations, as well as possible feedback mechanisms in which HIF-1α and VEGF upregulate EGLN 1 transcription (37, 38). Decreased EGNL 1 expression post-injury in the HFD-fed group may be secondary to changes in metabolic activity in HFD-fed mice post-injury or decreased VEGF activity. Regulatory factors governing PHD2 levels in an obese state warrant further research. Interestingly, at baseline levels, nuclear HIF-1α is significantly increased in mice fed HFD, but this does not correlate to increased VEGF levels. This may be secondary to decreased HIF-1α binding to its promoter or increased VEGF degradation at baseline in skeletal muscle. Following injury, however, there is instead a trend toward increased HIF-1α in mice fed a lean diet following injury, suggesting that the HFD-fed mice are unable to respond appropriately to skeletal muscle injury.

Muscle-specific knockout of HIF-1α results in extensive muscle damage and decreased endurance in response to exercise (39). Yet, in non-obese conditions, HIF-1α knockout in skeletal muscle influences neither the severity of acute myotrauma nor skeletal muscle regeneration, but knockout of HIF-1α in myeloid cells significantly delays muscle regeneration (40). In normoxic conditions, HIF-1α is found predominantly in the cytoplasm of skeletal muscle (41). The present study found no differences in the skeletal muscle HIF-1α nuclear levels, either following injury or with DMOG treatment. Instead, there were increased levels of HIF-1α in the cytoplasm of RD-fed mice as compared to HFD-fed mice following injury. The exact role and compartmental localization of HIF-1α in skeletal muscle regeneration in a model of obesity remains unclear and warrants further investigation. One possibility, supported by the data reported here, is that HIF-1α exerts its effect on muscle by promoting transcription of VEGF. Originally described as an endothelial-specific growth factor, recent evidence suggests that the effects of VEGF might extend to a variety of other cell types, including skeletal muscle (42). The present study is the first to suggest that VEGF is differentially regulated in skeletal muscle in response to obesity.

Although this study suggests a role for hydroxylases and the hypoxia pathway in skeletal muscle regeneration, there are further research areas that still need to be considered. DMOG can inhibit multiple prolyl hydroxylase isotypes; transgenic muscles with specific deletions will likely be necessary to identify specific subtypes involved. In addition, utilizing whole muscle lysates does not account for potential differences in immune cell infiltration following injury. Assessment of skeletal muscle precursors and their regulation by VEGF *in vitro* could further explain the role of VEGF in this pathway. In addition, muscle-specific loss of VEGF could further elucidate mechanisms related to regeneration, specifically with regard to obesity. It must be noted that the current study is limited to fast twitch muscle, and involvement of the hypoxia pathway in slow twitch muscles was not addressed here. Finally, although no differences were seen in HIF-2α levels between mice fed HFD or RD at day 5 after cryoinjury, it is possible that HIF-2α was differentially regulated at a separate time point and may still be involved. Other studies have suggested that transgenic or pharmacological inhibition of HIF-1α in adipocytes prevents the onset of obesity from overfeeding and improves insulin sensitivity (43). This suggests that the hypoxia pathway may have tissue specificity with regard to pathologic under or overexpression and requires further investigation.

Despite its limitations, this study identifies a potentially novel pathway in identifying skeletal muscle regeneration differences that occur with obesity. A more detailed understanding of the hypoxia pathway with regard to skeletal muscle regeneration may identify therapeutic, pharmacologic targets. Clinically, obese patients suffer a loss of skeletal muscle mass, and interventions are necessary to prevent excessive skeletal muscle loss in these patients.

## Ethics Statement

This study was carried out in accordance with the recommendations of the Institutional Animal Care and Use Committee of Brigham and Women’s Hospital and Joslin Diabetes Center, which also approved all animal procedures described (2016N000375).

## Author Contributions

IS drafted the manuscript; DS conducted the experiments; BO and JW helped design the experiments; CK, DV, and KN performed animal studies; DV and JS helped with the overall study and developed the figures; AM analyzed the data; and IS and AW interpreted the data and brainstormed ideas. All the authors contributed to intellectual content and critical revisions.

## Conflict of Interest Statement

The authors declare that the research was conducted in the absence of any commercial or financial relationships that could be construed as a potential conflict of interest.

## Footnotes

^1^Diet-induced Obesity Models. The Jackson Laboratory. Available from: https://www.jax.org/jax-mice-and-services/find-and-order-jax-mice/surgical-and-preconditioning-services/diet-induced-obesity-models/dio-b6.
