# Supplementary figures and images for: Prolyl Hydroxylase Domain-2 Inhibition Improves Skeletal Muscle Regeneration in a Male Murine Model of Obesity
Source: Front Endocrinol (Lausanne). 2017 Jul 5;8:153. doi: 10.3389/fendo.2017.00153 (PMC5497248; doi:10.3389/fendo.2017.00153)

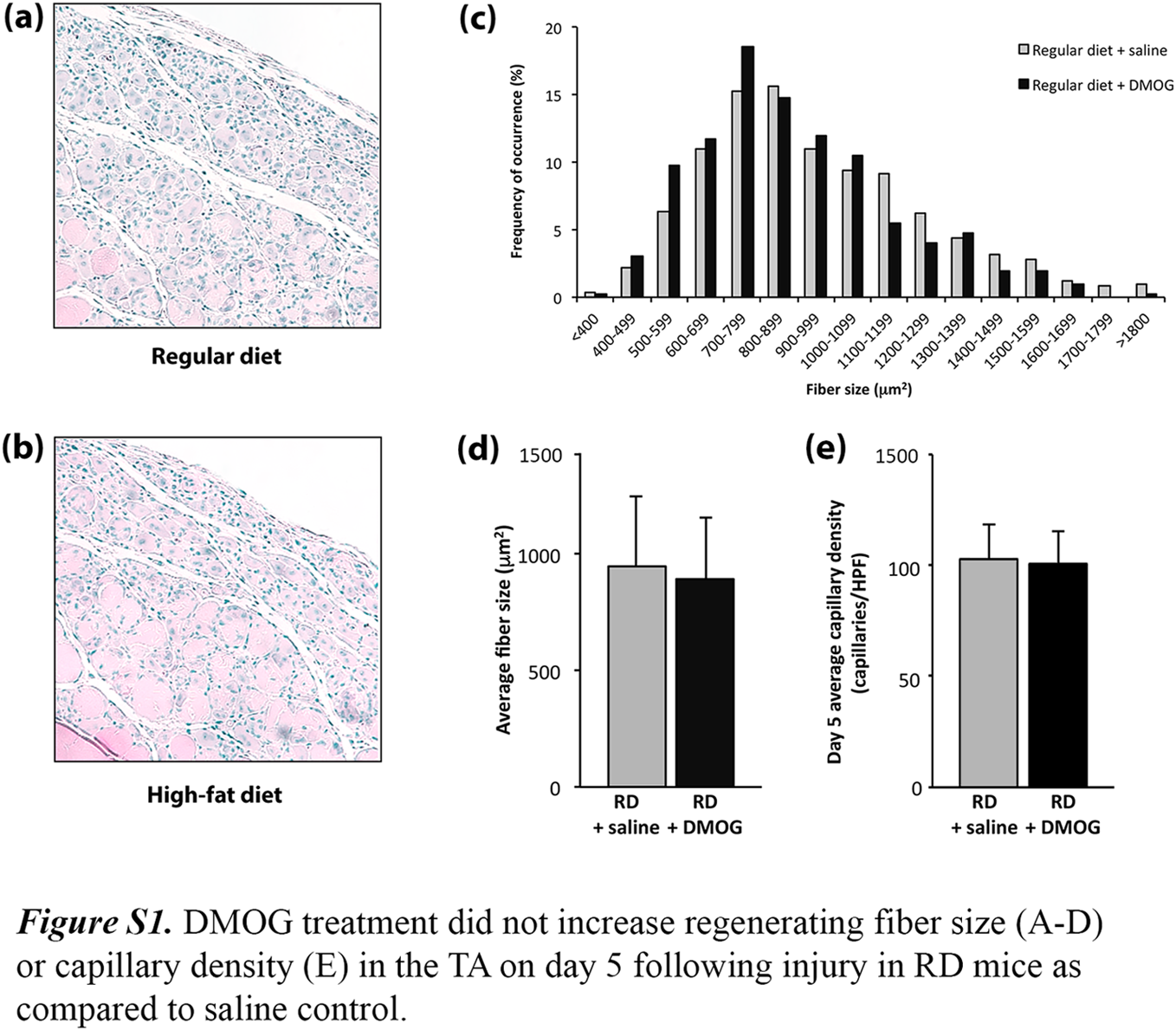

Supplement: Supplementary file 1 [file image_1.tif]

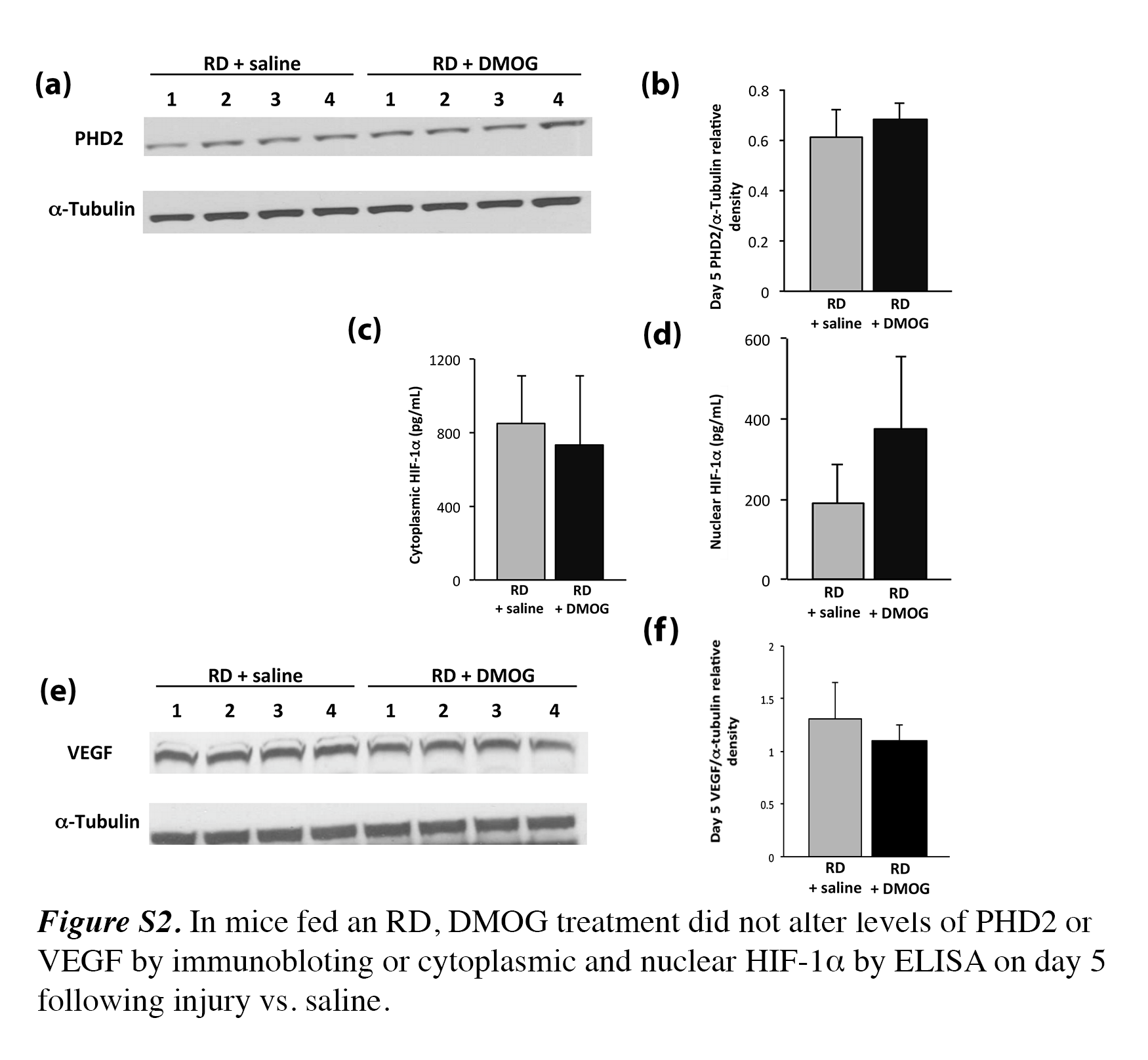

Supplement: Supplementary file 2 [file image_2.tif]
